# Quantifying bias in psychological and physical health in the UK Biobank imaging sub-sample

**DOI:** 10.1093/braincomms/fcac119

**Published:** 2022-05-09

**Authors:** Donald M. Lyall, Terry Quinn, Laura M. Lyall, Joey Ward, Jana J. Anderson, Daniel J. Smith, William Stewart, Rona J. Strawbridge, Mark E. S. Bailey, Breda Cullen

**Affiliations:** 1 Institute of Health and Wellbeing, University of Glasgow, 1 Lilybank Gardens, Scotland G12 8RZ, UK; 2 Institute of Cardiovascular and Medical Sciences, University of Glasgow, Scotland, UK; 3 Division of Psychiatry, University of Edinburgh, Edinburgh, Scotland, UK; 4 Department of Neuropathology, Queen Elizabeth University Hospital, Scotland, UK; 5 Cardiovascular Medicine Unit, Department of Medicine Solna, Karolinska Institutet, Stockholm, Sweden; 6 School of Life Sciences, College of Medical, Veterinary and Life Sciences, University of Glasgow, Glasgow, Scotland, UK

**Keywords:** epidemiology, psychological, imaging, bias, UK Biobank

## Abstract

UK Biobank is a prospective cohort study of around half-a-million general population participants, recruited between 2006 and 2010, with baseline studies at recruitment and multiple assessments since. From 2014 to date, magnetic resonance imaging (MRI) has been pursued in a participant sub-sample, with the aim to scan around *n* = 100k. This sub-sample is studied widely and therefore understanding its relative characteristics is important for future reports. We aimed to quantify psychological and physical health in the UK Biobank imaging sub-sample, compared with the rest of the cohort. We used *t*-tests and χ^2^ for continuous/categorical variables, respectively, to estimate average differences on a range of cognitive, mental and physical health phenotypes. We contrasted baseline values of participants who attended imaging (versus had not), and compared their values at the imaging visit versus baseline values of participants who were not scanned. We also tested the hypothesis that the associations of established risk factors with worse cognition would be underestimated in the (hypothesized) healthier imaging group compared with the full cohort. We tested these interactions using linear regression models. On a range of cognitive, mental health, cardiometabolic, inflammatory and neurological phenotypes, we found that 47 920 participants who were scanned by January 2021 showed consistent statistically significant ‘healthy’ bias compared with the ∼450 000 who were not scanned. These effect sizes were small to moderate based on Cohen’s *d*/Cramer’s *V* metrics (range = 0.02 to −0.21 for Townsend, the largest effect size). We found evidence of interaction, where stratified analysis demonstrated that associations of established cognitive risk factors were smaller in the imaging sub-sample compared with the full cohort. Of the ∼100 000 participants who ultimately will undergo MRI assessment within UK Biobank, the first ∼50 000 showed some ‘healthy’ bias on a range of metrics at baseline. Those differences largely remained at the subsequent (first) imaging visit, and we provide evidence that testing associations in the imaging sub-sample alone could lead to potential underestimation of exposure/outcome estimates.

## Teaser

UK Biobank is a general population cohort (0.5 million participants). From 2015 to date, magnetic resonance imaging has been pursued in a participant sub-sample. Lyall *et al*. demonstrate widespread bias towards better psychological and physical health in the imaging participants assessed so far (*N* ∼ 50 000).

## Graphical Abstract

**Graphical Abstract:**
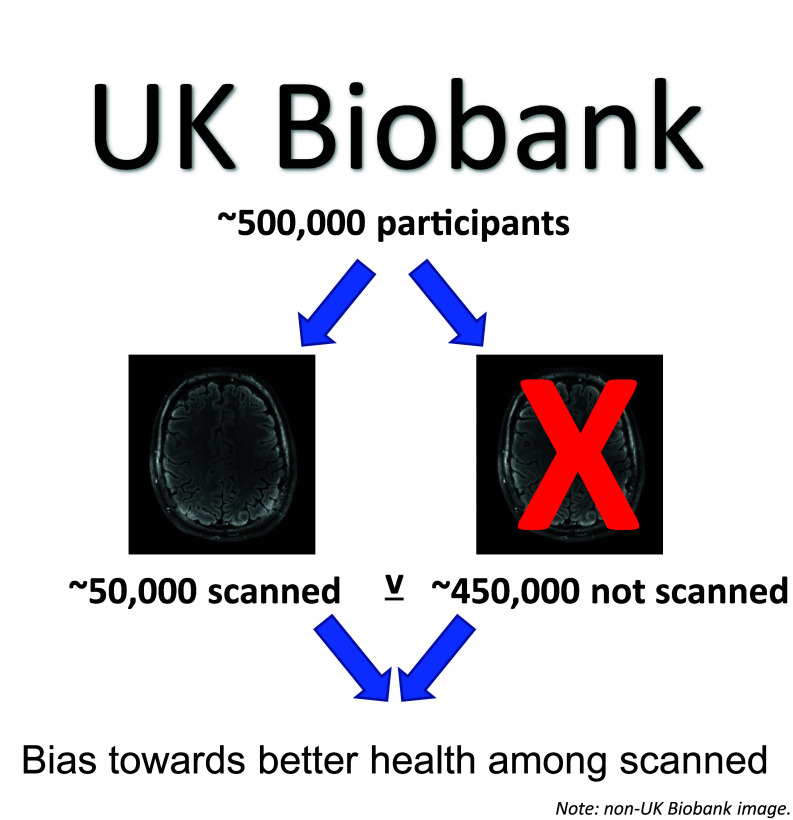

## Introduction

Understanding and where possible addressing the effects of bias and confounding are the fundamental aims of human epidemiology. UK Biobank is a relatively large population cohort of around 0.5 million middle-aged to older adults recruited as generally healthy volunteers from England, Scotland and Wales. UK Biobank’s 0.5 million participants reflect around a 5% response rate to initial invitations,^1^ with recognized recruitment bias where participants are relatively healthy (e.g. lower rates of smoking), well-educated and less deprived than the general UK population.^2^ This may have important implications for generalizability and extrapolating associations and exposure/outcome relationships to the broader population.

Most UK Biobank participants were between 40 and 70 years old at baseline assessment, which was completed from 2006 to 2010 when the mean age was around 60 years old. Since recruitment, a range of optional assessments has been offered/distributed to participants online via email (e.g. on diet preference, mental health, etc.).^1^ Further, from 2014, a sub-sample have been invited to attend magnetic resonance imaging (MRI) including of the heart, abdomen and brain, with the eventual aim being to recruit around 100 000 participants from UK Biobank to imaging. At these visits, most of the baseline questionnaires and clinical assessments are also repeated.^3^ In addition to the participation bias already noted in the cohort in baseline assessments at recruitment,^2^ it has been shown that there is further participation bias with regard to completing additional online assessments (versus attending only baseline) and that, to some extent, this participation bias has genetic contributions which vary by male/female sex.^4,5^

Previous studies of bias in the UK Biobank population have focused on participation at all, and/or predictors of additional participation in subsequent online assessments.^4,5^ We are not aware of research which has explored differences in participants who subsequently attended for imaging studies. Within each geographic region where UK Biobank scanning centres are located, all cohort members are invited via email and post to attend unless they have opted out of such communications or have left the UK (<0.5% of participants^3^). Nevertheless, it might be expected that those who go on to attend would differ systematically from those who do not. This could be for several reasons including MRI contraindications like stents and pacemakers, excluding less healthy participants, difficulties attending due to practical or health-related factors, and less healthy participants at baseline being more likely to die before imaging began.^6^

We asked whether those who underwent imaging were healthier on average at baseline, and whether they remained so by the time of imaging. We also tested for interactions to investigate if (eventual) imaged status was an effect measure modifier; if this were the case, studies using only the imaging sample might underestimate established exposure/outcome associations compared with the full 502k cohort. This report quantifies differences in key cognitive, mental and physical health metrics (chosen *a priori*) in the imaging sub-sample.

## Methods

### Study design and participants

UK Biobank is a large prospective cohort study including 502 490 general population participants who attended one of 22 baseline assessment centres from 2006 to 2010 where they completed a series of physical, sociodemographic and medical assessments.^1^ In 2014, MRI scanning of the heart, brain and abdomen for a sub-group of participants began, and this is ongoing with an eventual target sample size of 100 000. As of January 2021, MRI data were available on 47 920 participants who attended three centres (Cheadle, Newcastle and Reading) using identical protocols. An additional *n* = 1069 participants were not deemed safe (2%) and were not included in the ‘imaged’ group. This project was completed using UK Biobank project #17689. For all variables, we removed participants who chose not to answer/did not know (<5%).

### Ethical approval

This secondary-data analysis study was conducted under generic approval from the NHS National Research Ethics Service (approval letter dated 13 May 2016, Ref. 16/NW/0274). Written informed consent was obtained from all participants recruited to UK Biobank.

### Demographic data

Age, sex, ethnicity and educational attainment were self-reported. Townsend deprivation index was derived from postcode of residence.^7^ This provides an area-based measure of socioeconomic deprivation derived from aggregated data on car ownership, household overcrowding, owner occupation and unemployment. Higher Townsend scores equate to higher levels of area-based socioeconomic deprivation.

### Blood pressure and anthropometric data

Diastolic and systolic blood pressure (DBP; SBP) were assessed using digital blood pressure monitors (HEM-7015IT; Omron Healthcare Inc.). We used the second reading because there is evidence the first reading can overestimate blood pressure.^8^ Weight was measured, to the nearest 0.1 kg, using the Tanita BC-418 MA body composition analyser. Height was measured using a Seca 202 height measure. Body mass index (BMI) was derived as weight (kg)/[height (m)×height (m)] by UK Biobank centrally. Participants removed their shoes and heavy outer clothing before weight and height were measured.

### Cognitive data

We examined tests that were included as part of the UK Biobank baseline cognitive assessment.^9^ One of these was a task with 13 logic/reasoning-type questions and a 2-min time limit labelled ‘fluid intelligence’ in the UK Biobank protocol but hereafter referred to as *verbal-numerical reasoning*. The maximum score was 13 where higher scores indicate better performance. *Pairs matching* was a visuospatial memory test, where participants were asked to memorize the positions of six card pairs, and then match them from memory while making as few errors as possible. We refer to this test as the memory task from here on. Scores on the memory test are for the number of errors that each participant made, and higher scores are, therefore, worse. For the prospective memory (PM) test, participants were asked to engage in a specific behaviour later in the assessment: ‘At the end of the games we will show you four coloured symbols and ask you to touch the blue square. However, to test your memory, we want you to actually touch the Orange Circle instead’. We scored participants as zero or one, depending on whether they completed the task on first attempt or not. Participants completed a timed test of symbol matching similar to the common card game Snap, which we refer to as the reaction time (RT) task; scores are measured in milliseconds with higher values indicating worse performance. The PM and reasoning tests were added part-way through the baseline assessments and are therefore available only in around one-third of baseline participants but were completed by most at imaging.

### Psychological variables

Neuroticism was assessed using a point scale with values from 0 to 12, the Eysenck Personality Inventory Neuroticism scale—Revised.^10–12^ Depression (yes/no for lifetime history) was based on self-report. Participants self-reported their ‘happiness’ ordinally (extremely unhappy; very unhappy; moderately unhappy; moderately happy; very happy; extremely happy). This variable was added part-way through the baseline assessments and was completed then by around one-third of participants. We derived a dichotomous variable where participants were moderately/very/extremely unhappy versus not.

### Lifestyle

Participants self-reported their smoking history: current, past or never. We collated past and current smokers into ‘ever’ (versus never). Participants self-reported their alcohol intake frequency on a 5-point ordinal scale from ‘never’ to ‘daily or almost daily’. We derived a dichotomous variable (‘regular drinkers’ versus not) of collated ‘daily or almost daily’, ‘three or four times a week’ and ‘once or twice a week’ versus ‘one to three times a month’, ‘special occasions only’ and ‘never’. We excluded participants from this specific variable if they reported having changed their alcohol intake due to health problems or doctor’s advice.^13^

### Physical health conditions (cardiometabolic, inflammatory and neurological)

Using self-report, participants responded to the touch-screen question ‘Has a doctor ever told you that you have had any of the following conditions?’ (high blood pressure; stroke; angina; heart attack). We collated heart attack and angina into coronary heart disease (CHD). Participants were also asked ‘Has a doctor ever told you that you have diabetes?’ via touch-screen.^14^ During an interview with a trained staff member, participants noted their history of any other medical conditions, and from this we derived the phenotypes of any versus none for inflammatory conditions (e.g. rheumatoid arthritis), detailed in a previous open-access paper,^15^ and neurological conditions.^9^ Participants self-reported regular medication for cholesterol, blood pressure, diabetes and/or take exogenous hormones, from which we derived a dichotomous variable (any versus none). Participants self-reported their overall health as excellent; good; fair; poor. We derived two dummy variables: excellent/good versus fair (good health) and poor versus fair (poor health).

### Statistical analyses

We tested for unadjusted differences in baseline values (2006–2010) in people who had not been imaged as of January 2021, versus baseline values in people who had been imaged. We also contrasted baseline values in the non-imaged group (i.e. 2006–2010) versus values of imaged participants at the time of scan (2014–2020). Townsend did not receive repeat assessment at imaging, and some were only repeated in some participants so were excluded from analysis, e.g. ‘Has a doctor ever told you that you have had any of the following conditions?’ (https://biobank.ndph.ox.ac.uk/ukb/field.cgi?id=6150). For continuous variables, we used *T*-tests to derive *P*-values, and report Cohen’s *d* effect sizes (standardized mean difference where 0.2 is small, 0.5 is medium and 0.8 is large). Due to non-normal distributions, we natural log-transformed the memory variable scores (+1) and RT tests. For categorical variables, we used χ^2^ tests and Cramer’s *V* as a metric of standardized effect size (0.1 is small, 0.3 is medium and 0.5 is large). Given the duration between the baseline and imaging visits, as a secondary analysis, we adjusted the imaging-concurrent versus non-imaged baseline contrasts for age at time of assessment using linear/logistic regressions for continuous/binary variables, respectively.

Using baseline data from the whole cohort, we used linear regression to estimate associations between established risk factors and cognitive performance, including testing for interactions with imaged status. The established risk factors we tested were self-reported doctor diagnoses of CHD, diabetes and high blood pressure.^14^

A small number of participants who attended the MRI visit were deemed not safe to scan; these were classified in the non-imaged group in all analyses (in line with the fact that many other people in the non-imaged group would not have volunteered for scanning or would have been screened out prior to the visit because of known contraindications). We report uncorrected *P*-values throughout; as sensitivity analysis, we correct final results for type-1 error using false discovery rate (FDR).^16,17^

### Data availability statement

UK Biobank is an open-access resource available to verified researchers upon application (http://www.ukbiobank.ac.uk/). Analysis syntax is available from the Open Science framework: https://osf.io/zu8xm/.

## Results

At baseline recruitment to UK Biobank, there were *n* = 502 490 participants, with an overall mean age = 56.53 [standard deviation (SD) = 8.10] and 229 114 (45.60%) were males. Of those baseline participants, *N* = 48 989 attended MRI by date of analysis, of which *N* = 47 920 went on to be scanned, representing the ‘MRI’ sub-sample. The mean age of the MRI sub-sample at time of scan was 64.15 (SD = 7.73) with the typical interval between baseline and imaging 8.98 (minimum 4, maximum 14, SD = 1.8) years.

### Baseline visit values

We first contrasted baseline values of people who were later imaged versus not. The imaged group showed consistently significant differences for lifestyle, psychological and cardiometabolic and demographic variables (see Table 1). The metrics of standardized effect size (Cohen’s *d* for continuous variables; Cramer’s *V* for dichotomous) varied from 0.02 for sex to −0.21 for Townsend; i.e. there was a statistically significant but effectively very small bias towards being female, and a larger but still small bias towards less deprivation in the imaged group. Other notable but still ultimately small effects were seen for younger age and lower BMI. In terms of psychological variables, all variables showed statistically significant differences, which ranged from Cohen’s *d* = −0.08 (log visual memory errors) to reasoning (0.36). Overall, when compared with non-imaged participants at baseline, participants who went on to be imaged were: slightly younger (average 55 versus 57 years); more likely to be female; more likely to have a degree; White British; less deprived; at lower risk of diabetes, hypertension, CHD, inflammatory and neurological conditions; less likely to self-report medication usage; have ever-smoke; had lower BMI and DBP/SBP, lower risk of depression, lower neuroticism and show better cognitive scores. Most of these effect sizes were relatively small. Descriptive statistics are shown in Supplementary Table 1.

**Table 1:** Imaged versus non-imaged baseline values: demographic, lifestyle, psychological and physical health variables

					95% CI for mean difference		
	*t*	df	*P*-value	Mean difference	Lower	Upper	Cohen's *d*
Age (years)	−38.724	502 488	<0.001	−1.503	−1.579	−1.427	−0.186
Sex (male N*)	132.469	1	<0.001	−0.111	−0.129	−0.092	0.016
Degree yes, N*	4365.962	1	<0.001	−0.635	−0.654	−0.616	0.094
White British, N*	416.475	1	<0.001	−0.342	−0.375	−0.309	0.029
Townsend score	−43.035	501 865	<0.001	−0.639	−0.668	−0.610	−0.207
Diabetes, N*	668.37	1	<0.001	0.724	0.668	0.781	−0.037
High blood pressure, N*	1214.326	1	<0.001	0.41	0.387	0.433	−0.049
Coronary heart disease, N*	567.799	1	<0.001	0.714	0.654	0.774	−0.034
Neurological condition, N*	328.033	1	<0.001	0.509	0.454	0.565	−0.026
Inflammatory condition, N*	631.573	1	<0.001	0.356	0.328	0.384	−0.036
Medication use, N*	740.545	1	<0.001	0.39	0.362	0.418	−0.038
Smoking status (ever smoker), N*	672.739	1	<0.001	0.254	0.235	0.273	−0.037
Alcohol frequency (≥weekly), N*	970.125	1	<0.001	−0.36	−0.383	−0.338	0.045
Diastolic blood pressure (mmHg)	−14.125	461 276	<0.001	−0.729	−0.830	−0.628	−0.071
Systolic blood pressure (mmHg)	−27.554	461 272	<0.001	−2.581	−2.764	−2.397	−0.138
Body mass index	−38.317	499 384	<0.001	−0.883	−0.929	−0.838	−0.184
Good/excellent health, N*	1803.851	1	<0.001	−0.572	−0.599	−0.546	0.062
Poor health, N*	242.253	1	<0.001	0.571	0.498	0.644	−0.043
Verbal-numerical reasoning score	43.867	165 452	<0.001	0.782	0.747	0.817	0.364
Log reaction time	−40.482	496 663	<0.001	−0.037	−0.039	−0.035	−0.195
Reaction time (ms)	−39.888	496 663	<0.001	−22.612	−23.724	−21.501	−0.192
Log visual memory errors	−15.956	497 865	<0.001	−0.051	−0.057	−0.045	−0.077
Visual memory errors	−20.894	497 865	<0.001	−0.343	−0.375	−0.311	−0.101
Prospective memory, successful on first attempt, N*	432.074	1	<0.001	−1.13	−1.243	−1.018	0.056
Unhappy, N*	18.859	1	<0.001	0.18	0.099	0.262	−0.01
Neuroticism score	−19.933	401 561	<0.001	−0.342	−0.375	−0.308	−0.105
Self-reported depression, N*	78.898	1	<0.001	0.199	0.155	0.243	−0.013

Student’s *t*-test for continuous variables. For dichotomous variables denoted by N*: *t* = chi-square *x^2^*, mean difference = log odds ratio (difference), Cohen’s *d* = Cramer’s *V*. Mean difference reflects imaged group (i.e. imaged average—non-imaged average).

### Imaging visit values

Where possible we repeated the previous analyses but contrasting imaging-concurrent values versus non-imaged participant baseline data. These are shown in Table 2, with descriptive statistics in Supplementary Table 2. Excluding age, which had a large effect due to being several years later, effect sizes were largely similar. All tests were statistically significant at *P* < 0.05 except for diabetes prevalence (*P* = 0.628). The largest effect sizes were found for DBP (Cohen’s *d* = −0.36; lower in imaging group), reasoning (*d* = 0.32; higher/better in imaging group) and (log) RT (*d* = 0.34; slower in imaging group). For depression, neurological and inflammatory conditions, the directions of association changed where the imaging group had slightly higher rates. After adjusting additionally for age at time of assessment, all associations remained statistically significant to very similar degrees, except for ‘unhappy’ (versus happy; *P* = 0.982; Supplementary Table 3).

**Table 2:** Comparison of baseline values in the imaged versus non-imaged groups

					95% CI for mean difference		
	*t*	df	*P*-value	Mean difference	Lower	Upper	Cohen's *d*
Age (years)	192.182	502 488	<0.001	7.475	7.398	7.551	0.923
Diabetes, N*	0.235	1	0.628	−0.01	−0.051	0.031	0.001
Inflammatory condition, N*	163.087	1	<0.001	−0.156	−0.18	−0.132	0.018
Neurological condition, N*	1458.624	1	<0.001	−1.035	−1.091	−0.98	0.057
Medication use, N*	10.149	1	0.001	0.041	0.016	0.066	−0.005
Smoking status (ever smoker), N*	1128.722	1	<0.001	0.333	0.313	0.352	−0.048
Alcohol frequency (≥weekly), N*	4546.902	1	<0.001	−0.903	−0.93	−0.876	0.097
Diastolic blood pressure (mmHg)	−66.757	455 124	<0.001	−3.699	−3.808	−3.591	−0.359
Systolic blood pressure (mmHg)	5.115	455 116	<0.001	0.515	0.318	0.713	0.028
Body mass index	−40.829	497 530	<0.001	−0.959	−1.005	−0.913	−0.200
Good/excellent health, N*	994.52	1	<0.001	−0.407	−0.433	−0.382	0.046
Poor health, N*	247.755	1	<0.001	0.536	0.468	0.603	−0.044
Verbal-numerical reasoning score	58.665	193 423	<0.001	0.680	0.657	0.703	0.318
Log reaction time	68.365	493 482	<0.001	0.065	0.063	0.067	0.339
Reaction time (ms)	59.827	493 482	<0.001	35.251	34.096	36.406	0.297
Log visual memory errors	−26.991	495 485	<0.001	−0.088	−0.095	−0.082	−0.134
Visual memory errors	−30.531	495 485	<0.001	−0.513	−0.545	−0.480	−0.151
Prospective memory, successful on first attempt, N*	47.545	1	<0.001	−0.168	−0.216	−0.12	0.017
Unhappy, N*	133.65	1	<0.001	0.316	0.262	0.37	−0.026
Neuroticism score	−67.671	408 823	< 0.001	−1.08	−1.112	−1.049	−0.33
Self-reported depression, N*	31.989	1	<0.001	−0.112	−0.151	−0.073	0.008

Student’s *t*-test for continuous variables. For dichotomous variables denoted by N*: *t* = chi-square *x^2^*, mean difference = log odds ratio (difference), Cohen’s *d* = Cramer’s *V*. Mean difference reflects imaged group (i.e. imaged average—non-imaged average).

### Interaction tests: imaging status and established risk factors versus cognitive scores

We tested the hypothesis of interaction between imaged status and known risk factors for baseline cognitive scores, cross-sectionally in the whole cohort. Models included continuous age, sex, imaged status, (risk factor) and [(risk factor) × imaged status]. The models were run separately for log RT, log memory errors and reasoning scores.

Overall, there were several interactions whereby the imaged group showed significantly smaller associations between established cardiometabolic risk factors and baseline cognitive scores, compared with the associations in the non-imaged group.

For CHD, interactions with imaged status were statistically significant for log RT and reasoning (*P* < 0.001) but not log memory errors (*P* = 0.559); indicative of differential associations of risk factors with cognitive abilities depending on whether participants were later imaged or not. The standardized beta estimate (*β*), reflecting differences in SD units for CHD versus log RT was significantly larger in the non-imaged sample (*β* = 0.026, *P* < 0.001) versus imaged group (*β* = 0.009, *P* = 0.046). This was also the case for CHD and reasoning (*β* = −0.047, *P* < 0.001 non-imaged versus −0.039, *P* < 0.001 imaged).

For diabetes, there were significant interactions for log RT and reasoning (both *P* < 0.001) but not log memory errors (*P* = 0.095). For log RT the association was larger in non-imaged (*β* = 0.040, *P* < 0.001) versus imaged (0.020, *P* < 0.001) and similarly for reasoning: larger in non-imaged (−0.054, *P* < 0.001) compared with imaged (−0.019, *P* = 0.014).

For high blood pressure, the interaction terms were significant in all three models (*P* < 0.001): log RT (non-imaged *β* = 0.029, *P* < 0.001 versus imaged *β* = 0.016, *P* < 0.001); log memory errors (non-imaged *β* = 0.005, *P* = 0.001 versus imaged β = 0.002, *P* = 0.697); and finally reasoning (non-imaged β = −0.052, *P* < 0.001 versus imaged −0.028, *P* = 0.001).

### Sensitivity analyses

We determined the BP results were unchanged when we added 10/15 mmHg to DBP/SBP, respectively,^18^ for participant baseline/imaging values if they also reported antihypertensive medication. No association *P*-values attenuated when corrected for multiple testing with FDR. Study results were unchanged when we included the participants who volunteered for but did not complete imaging, in either the imaging and/or non-imaged groups. In addition to simply ‘completed MRI’, we restricted analysis to participants with useable data (based on https://biobank.ndph.ox.ac.uk/showcase/field.cgi?id=25767) which lowered sample size from *N* = 47 920 to *N* = 32 778. Findings were very similar, with effect sizes generally agreeing to the second or third decimal point (e.g. for reasoning the initial Cohen’s *d* = 0.36 became 0.37). Results were unchanged when we removed outliers 4SDs from respective value means.

## Discussion

This study aimed to quantify and characterize differences between the sub-sample of approximately 50 000 UK Biobank participants who had completed imaging as of January 2021 and the wider UK Biobank cohort. Overall, our data demonstrate a degree of ‘healthy bias’ among imaged participants when compared with non-imaged participants with regards a range of psychological, cardiometabolic, cognitive, inflammatory, lifestyle and demographic variables. Participants who were subsequently imaged for the first time were shown to have healthier demographics and lifestyles (e.g. lower deprivation and smoking history), better psychological health (less depression; unhappiness; lower neuroticism), better cognitive abilities (memory; reasoning; information processing speed) and physical health (lower prevalence of several different conditions; lower BP). This has value with regard to understanding selection biases in a cohort already appreciated to have bias towards healthier individuals in the population and a restricted range on some key demographic variables.

The effect sizes for these differences were generally small to moderate. When we compared non-imaged participant baseline values (2006–2010) to measures taken at imaging (2014 to date) these effect sizes were generally consistent with those observed cross-sectionally at baseline. Exceptions included slightly increased rates of depression, chronic inflammatory conditions and that the imaging group performed worse on the RT test. This survived correction for age at time of assessment. It is not clear why: this may reflect test imprecision, non-linear biological/cognitive ageing and accumulation not captured by the age at time of assessment value, and/or systematic differences in procedure at baseline versus MRI, for example that the imaging visit cognitive assessment was longer, including tests not reported here.^6^ This added length may contribute to fatigue. Reasoning scores remained better than the baseline group, which may reflect the presence of items in the test served by accumulated ‘crystallized’ intelligence.^9^

### Implications

Volunteer bias in UK Biobank is recognized, in terms of participation at all versus the general population, as well as between baseline and later optional online assessments.^2,5^ It has been noted that this raises the possibility of collider bias, which can distort estimates of exposure/outcome associations either towards or away from the null.^19^ This report documents additional differences between the sub-group of UK Biobank participants who were subsequently imaged compared with those who were not as of January 2021. We show that if researchers were to analyse a known association^14^—here cardiometabolic conditions versus cognitive abilities—using only the imaging sub-sample, there is a risk they would underestimate the true magnitude of association. This supports the assertion that non-representativeness and selection bias can have a meaningful impact on interpretation and estimation of effect size.^20^ Where possible, researchers should incorporate data from the full UK Biobank cohort, seek replication cohorts and acknowledge and adjust for potential healthy bias and restrictions of range.^21^ Methods for adjustment include post-stratification, raking, calibration, raking with lasso variable selection, regression for estimating response propensity and Bayesian additive regression trees (BART) for estimating response propensity and raking, where there is evidence BART is most effective.^22^

### Limitations and future research

This study used data from around 50k participants with MRI data as of January 2021. The ultimate expectation is that 100k participants will undergo MRI scanning, and it will be informative to re-test these variables in that larger sample to see whether the biases identified here persist as the data set grows. Future studies may investigate more fine-grained analyses, e.g. use of fine-grained psychotropic medication history as a proxy for psychological health, and/or individual conditions rather than collated sets as were used here in some instances. A sub-sample of the imaging cohort is undergoing longitudinal scanning; investigation of bias in that group is important to consider, and whether there is differential bias according to certain key variables like proximity to assessment centres.

## Conclusion

UK Biobank is a relatively large prospective research cohort which has been used extensively in recent years to investigate exposure/outcome associations at scale, across a wide variety of fields including psychiatry, cognitive ageing, dementia, inflammation, immunity, cancer and cardiology.^23^ Since 2014, UK Biobank has assessed a sub-sample of the original 502k participants with MR imaging, with the aim of scanning 100k participants including some longitudinally.^24^ Here we show evidence for a small to moderate degree of healthy bias at baseline which mostly persisted at the time of imaging itself, when comparing participants who were scanned with those who were not (as of January 2021). This is over and above the healthy bias already present in the whole cohort at baseline compared with the general population. We show that testing exposure/outcome associations using only the imaging sample would lead to a significant underestimate of effect, which has important implications for the planning and interpretation of MRI studies in UK Biobank.

## Supplementary Material

fcac119_Supplementary_Data

## Abbreviations

BART =: Bayesian additive regression trees
CHD =: coronary heart disease
DBP =: diastolic blood pressure
MRI =: magnetic resonance imaging
PM =: prospective memory
RT =: reaction time
SBP =: systolic blood pressure
